# Tobacco Use among School-Going Adolescents in Comoros: A Secondary Analysis of the 2015 Comoros Global Youth Tobacco Survey

**DOI:** 10.1155/2022/7134340

**Published:** 2022-03-08

**Authors:** Peter Bai James, Said Abasse Kassim, John Alimamy Kabba, Chenai Kitchen

**Affiliations:** ^1^National Centre for Naturopathic Medicine, Faculty of Health, Southern Cross University, Lismore, New South Wales 2480, Australia; ^2^Faculty of Pharmaceutical Sciences, College of Medicine and Allied Health Sciences, University of Sierra Leone, Freetown, Sierra Leone; ^3^Département de Management, Centre de Recherche en Gestion Des Services de Sante, Faculté Des Sciences de l'administration (FSA), Université Laval (UL), Centre Hospitalière Universitaire (CHU) de Québec, Québec, Canada G1V 0A6; ^4^Department of Pharmacy Administration and Clinical Pharmacy, School of Pharmacy, Xi'an Jiaotong University, #76 Yanta West Road, Xi'an 710061, China

## Abstract

**Background:**

Tobacco use among adolescents has long-term adverse health consequences, especially during adulthood. Currently, little is known about tobacco use behaviour among adolescents in Comoros. Our study aims at estimating the prevalence and identifying key factors associated with tobacco use among adolescents in Comoros using the 2015 Comoros Global Youth Tobacco Survey data.

**Methods:**

A national cross-sectional survey secondary data of 2,810 eligible school-going adolescents aged between 11 and 17 years was analysed. Complex sample logistic regression analyses were used to determine the correlates of current cigarette smoking and current use of noncigarette tobacco products.

**Results:**

The overall prevalence of current cigarette smoking was 14.3% [males (18.5%) and females (9.9%)]. The prevalence of current use of noncigarette tobacco products was 5.8% [males (6.7%) and females (4.9%)]. Being male (AOR = 2.24; 95% CI:1.39-3.63), exposure to secondhand smoke inside (AOR =3.88; 95% CI:2.84-5.31) and outside (AOR =1.49; 95% CI: 1.08-2.03) their home, and exposure to tobacco industry promotion (AOR =2.90; 95% CI:2.21-3.80) were associated with current cigarette use among school-going adolescents. However, parental smoke (AOR = 1.20; 95% CI:0.78-1.87) and not exposed to antismoking education in schools (AOR = 0.97; 95% CI:0.76-1.22) were not associated with current cigarette use. On the other hand, being male (AOR = 1.24; 95% CI:0.82-1.86) was not associated with the current use of noncigarette tobacco products. Adolescents who were exposed to tobacco industry promotion (AOR = .2.58; 95% CI:1.54-4.32) and not exposed to antismoking education in school (AOR = 0.52; 95% CI:0.32-0.85) were more and less likely associated with noncigarette tobacco use.

**Conclusion:**

One in seven school-going adolescents smokes cigarettes, and approximately one in 20 school-going adolescents uses noncigarette tobacco products in Comoros. Exposure to secondhand smoke within and outside the home and exposure to tobacco industry promotion were associated with tobacco use in school-going adolescents in Comoros. Our findings suggest the need for adolescent-friendly gender-sensitive tobacco interventions, including strengthening existing tobacco control laws to prevent and reduce tobacco use among school-going adolescents in Comoros.

## 1. Background

Globally, tobacco use is considered a significant risk factor for noncommunicable diseases, including nicotine addiction, and a means through which youths are introduced to using other illegal substances [1–3]. In recent years, low- and middle-income countries have started experiencing the public health impact of noncommunicable diseases related to the increased use of tobacco products [4]. This increased utilisation rate has been attributed to the influx of transnational tobacco companies in these countries due to weak tobacco legislations and policies, low levels of education, and demographic shift in the population in these areas [5, 6]. Studies conducted in African countries have reported increasing use of tobacco products among adolescents [7–9]. Factors such as being male, exposure to secondhand smoking within and outside the home, peer influence, and exposure to tobacco industry promotion, advertisement, and sponsorship have been associated with the use of tobacco products among adolescents [7–10].

Comoros is an archipelago located in the Indian Ocean, north of the Mozambique Channel, and northeast Madagascar and composed of three main islands (Grande Comore, Anjouan, and Mohéli) [11], Comoros is a developing country with 25% of poor population below the country's poverty line and is known to have poor health outcomes such as high maternal and child mortality compared to its neighbouring island nations of Mauritius and Seychelles [12, 13]. In 2018, one in five adults in Comoros used tobacco, which creates a future public health threat [14]. This public health threat is compounded by the fact that noncommunicable disease is a major public health concern in Comoros [15, 16]. In 2008, 25.4% of the population had high blood pressure, 4.8% had diabetes, and 25.9% had high cholesterol levels [15]. Death due to noncommunicable diseases has increased from 42% in 2015 to 45% in 2019 [17]. Similar to other African countries, Comoros, in 2006, became a signatory to the World Health Organization (WHO) Framework Convention on Tobacco Control (FCTC). In line with the WHO-FCTC framework, Comoros enacted three laws since 2010 that regulate tobacco product use [18]. These regulations include the use of smoke-free places, tobacco advertising, promotion and sponsorship, and tobacco packaging and labelling [18]. Despite the impact of these laws in reducing tobacco use prevalence from 30.4% in 2007 to 19.5% in 2018, tobacco use remains high [14]. This relatively high use may be due to ambiguity in the interpretation of some of the laws. For example, even though smoking is banned in all public places, the legal instruments are hard to explain due to discrepancies and conflicting provisions in the law [18]. Another reason for the high use of tobacco may be due to nonadherence to the WHO- FCTC recommendation. For instance, Comoros currently levies a 25% excise tax on the retail price of tobacco products, which is far below the WHO tobacco excise taxes recommended threshold of 70% on all the retail price of tobacco products [18].

Given that most adult smokers initiate smoking during adolescence, it highlights the need to understand the tobacco use behaviour among adolescents [5]. Currently, a plethora of tobacco use research in Comoros have focused on adults [15], with little or no research on adolescents who account for more than half of the population [13]. Therefore, this study aims at estimating the prevalence and identifying key factors associated with tobacco use among adolescents in Comoros using data obtained in the 2015 Comoros Global Youth Tobacco Survey. An understanding of tobacco use behaviour among adolescents will inform public health policies and interventions designed to prevent adolescents' tobacco use initiation and transition into future adult smokers.

## 2. Methods

### 2.1. Study Design and Comoros Global Youth Tobacco Survey

We used data obtained from the Comoros Global Youth Tobacco Survey (GYTS) administered in 2015 by the Ministry of Health. The survey collected tobacco use information from 2,810 eligible school-going adolescents aged between 11 and 17 years. GYTS uses a global standardised methodology that measures and tracks key tobacco control indicators [19]. The survey is designed to obtain relevant information on tobacco use prevalence and its associated determinants such as secondhand smoke (SHS), pro and antitobacco media and advertising, access to and availability of tobacco products, and knowledge and attitudes regarding tobacco use. Details of the survey methodology have been described in previous studies [19]. Basically, the survey methodology involves a two-stage sample design in which schools are chosen with a probability proportional to the enrolment size in the first stage. In the second stage, classes are selected randomly within these schools. Students in the selected classes were eligible and had an equal chance of being chosen. The overall response rate was 83.8% [20]. This study adheres to STROBE guidelines for cross-sectional studies (Supplementary file 1).

### 2.2. Study Measures

Two outcome measures were used to measure tobacco use among adolescents in Comoros. These include current cigarette smoking and the current use of noncigarette tobacco products. Current cigarette smoking status was determined by respondents' choice of one or more days with question, “During the past 30 days, on how many days did you smoke cigarettes?” Current use of noncigarette tobacco products was determined by recording the respondent choice on any of the following two questions–“During the past 30 days, did you use any form of smoked tobacco products other than cigarettes (e.g., cigars, water pipes, cigarillos, little cigars, pipes)?” and “During the past 30 days (one month), did you use any form of smokeless tobacco products (e.g., chewing tobacco, snuff, dip)?”. The following measures were considered as independent factors based on the available literature [7–9, 21]. These factors were constructed from 28 questions. This includes age, sex, average spending per week, parental or peer smoking, exposure to secondhand smoke inside or outside the home, exposure to smoking or antismoking media messages, exposure to tobacco industry promotions, perceptions about smoking, and knowledge about harmful effects of smoking and SHS as well as attitudes toward smoking ban [20].  Table 1 gives further details of how the dependent and independent variables were defined and dichotomized in this study.

### 2.3. Ethical Consideration

No ethics approval was required for this study since it is a secondary analysis of the Comoros Global Youth Tobacco Survey (GYTS) dataset, which is available in the public domain (Available at https://extranet.who.int/ncdsmicrodata/index.php/catalog/123). Ethical procedures were conducted by institutions that funded, commissioned, and managed the survey prior to collecting the primary data, and that includes the Ministry of Health Comoros. Informed consent was obtained from the school authorities involved in the survey and the parents and guardians of adolescents enrolled in the survey. All data were anonymized prior to the authors receiving the data for secondary analysis. A preprint of this study has previously been published [22].

### 2.4. Data Analysis

We used the Statistical Package for Social Science (IBM, SPSS 27.0) to analyse our data. To estimate the prevalence of current cigarette smoking and current use of noncigarette tobacco products, we reported weighted data to adjust for sampling design effect and missing values due to nonresponses at school, class, and student levels. All analyses (descriptive and inferential) were conducted using complex sample icon on SPSS to account for complex sampling design and for incorporating weights. Due to established gender differences regarding tobacco use, we conducted separate analyses for male and female adolescents. We employed complex sample logistic regression analyses to determine the correlates of current cigarette smoking and current use of noncigarette tobacco products. We reported an adjusted odds ratio (AOR) and 95% confidence interval (CI). A *p* value less than 0.05 (*p* value < 0.05) was considered statistically significant. We used the Hosmer and Lemeshow test to test the fitness of our models. Hosmer and Lemeshow test for current cigarette smoker was (*p* = 0.212) and for current user of noncigarette tobacco products was *p* = 0.497. All models were checked for possible multicollinearity using variance inflation factor (VIF). The multicollinearity test for the explanatory variables for current cigarette smokers was mean VIF = 1.096, Min VIF = 1.017, and Max VIF = 1.239 and current user of noncigarette tobacco products was mean VIF = 1.093, Min VIF = 1.016, and Max VIF = 1.238.

## 3. Results

### 3.1. Characteristics of the Study Population

Two thousand eight hundred and ten school-going adolescents took part in the study. Close to half of them were males (*n* = 1310, 48.1%) and more than half were between the age of 13 and 15 years (*n* = 1551, 55.3%). Close to a quarter were exposure to parental smoking (*n* = 584; 21.2%). More than a quarter (*n* = 793, 28.8%) and close to two-thirds (*n* = 1636, 59.6%) were exposed to secondhand smoke in and outside their homes, respectively. Please see Table 2 for details.

### 3.2. Prevalence of Current Cigarette and Noncigarette Tobacco Product Use

Table 3 describes the prevalence of current cigarette smoke and noncigarette tobacco product use among school-going adolescents in Comoros based on the 2015GYTS. The overall prevalence of current cigarette smoking was (*n* = 400, 14.3%) with a significant difference between males (*n* = 246, 18.5%) and females (*n* = 143, 9.9%). The prevalence of current cigarette smoking was highest among adolescents exposed to secondhand smoke outside the home (*n* = 305, 78.2%), followed by those who favoured the smoking ban (*n* = 284, 76.6%). A similar pattern was observed among male students. Among females, the prevalence of current cigarette smoking was highest among those with income (*n* = 106, 74.1%), followed by exposure to secondhand smoke outside the home (*n* = 101, 72.7%).

The overall prevalence of current noncigarette tobacco product use was (*n* = 145, 5.8%) with little difference between males (*n* = 81, 6.7%) and females (*n* = 64, 4.9%). The highest prevalence of current use of noncigarette tobacco products was observed among those who were in favour toward smoking ban (*n* = 111, 78.6%) and this was the same among males and females. The least prevalence was observed among students aged 12 years and below (*n* = 7, 5.8%) followed by those exposed to SHS outside the home (70.3%). A similar pattern was observed among males and females (See Table 3 for details).

### 3.3. Determinants of Current Cigarette Smoking and Noncigarette Tobacco Product Use among Male and Female Adolescents in Comoros

Table 4 shows determinants of current cigarette smoking and noncigarette tobacco product use among male and female adolescents in Comoros. Males were two times more likely to be current cigarette smokers compared to females (AOR = 2.24; 95% CI:1.39-3.63). Adolescents aged 13-15 years (AOR = 3.11; 95% CI:1.53-6.35) and 16 and above (AOR = 4.89; 95% CI: 2.56-9.37) were more likely to be current cigarette smokers than those 12 years and younger. A similar association was observed among males but not females. Adolescents exposed to secondhand smoke inside (AOR = 3.88; 95% CI:2.84-5.31)) and outside (AOR = 1.49; 95% CI: 1.08-2.03) their home were more likely to be current cigarette smokers compared to those who were not exposed. Similarly, exposure to secondhand smoke in the home was a determinant of current cigarette smoking among males (AOR = 4.90; 95% CI: 3.95-6.08) and females (AOR = 2.89; 95% CI: 1.35-6.19). However, exposure to secondhand smoke outside the home was a determinant of current cigarette smoking only among males (AOR = 1.72; 95% CI: 1.20-2.46) but not females (AOR = 1.13; 95% CI: 0.60-2.13). Exposure to tobacco industry promotion was found to be associated with current cigarette smoking among adolescents in general (AOR = 2.90; 95% CI: 2.21-3.80), and this was the case for both males (AOR = 2.66; 95% CI: 1.86-3.82) and females (AOR = 3.44 (; 95% CI: 1.92-6.17). However, parental smoke (AOR = 1.20; 95% CI: 0.78-1.87) and not exposed to antismoking education in schools (AOR = 0.97; 95% CI: 0.76-1.22) were not associated with current cigarette use.

Adolescents exposed to tobacco industry promotion were more likely to be current users of noncigarette tobacco products than those not exposed to tobacco industry promotion (AOR = .2.58; 95% CI: 1.54-4.32). Similar relationship between exposure to tobacco industry promotion and noncigarette tobacco use was observed among males (AOR = 2.58; 95% CI: 1.39-4.78) and females (AOR = 2.54; 95% CI: 1.24-5.22). Adolescents who did not receive antismoking education in schools were 52% less likely to be current noncigarette tobacco users (AOR = 0.52; 95% CI: 0.32-0.85) than those not exposed to antismoking education in school. Among males, being knowledgeable about the harmful effects of smoking and SHS was a determinant of current use of noncigarette tobacco products (AOR = 1.75; 95% CI:1.03-2.99). (See Table 4 for details).

## 4. Discussion

This is the first nationally representative study of school-going adolescents in Comoros regarding their tobacco use behaviour (cigarette smoking and use of noncigarette tobacco products). We found that 14.3% of school-going adolescents in Comoros were smoking cigarettes, with a higher prevalence observed in males than females. Our prevalence was higher than those reported in studies conducted in Ghana [7], Malawi [23], and East African countries (Sudan and South Sudan, Kenya, Tanzania, and Ethiopia) [21, 24]. On the other hand, our prevalence of current cigarette smoking was lower than the rates reported in Madagascar [9], Ville du Sud, Cote d'Ivoire [25] and, Southeast Nigeria [26]. In addition, 5.8% of school-going adolescents in Comoros were current users of noncigarette tobacco products, with slight difference users observed among males than females. Our rate is lower than the rates reported in similar studies conducted in Madagascar [9], the Republic of Congo [8], Sudan, and South Sudan [21] but higher than the prevalence reported in a study conducted in Ilala Municipality, Tanzania [27]. Our findings suggest the need for youth-friendly tobacco control policies and interventions that are gender sensitive. Even though the prevalence of noncigarette tobacco products is lower than cigarette smoking, noncigarette tobacco products such as cigars and pipes are equally harmful as cigarettes. Thus, it is important that, in addition to cigarettes, the adverse effects of noncigarette tobacco products should be part of tobacco control interventions such as public education and advocacy strategies targeting adolescents in Comoros.

Our findings indicate a high level of tobacco use among adolescents exposed to secondhand smoke both within and outside the home in Comoros. Adolescents exposed to secondhand smoke inside and outside the home were more likely to be current cigarette smokers, observed in both males and females. In contrast, exposure to secondhand smoke inside and outside the house was not significantly associated with using noncigarette tobacco products. Our findings are in line with a similar study conducted in Madagascar, Sudan, and South Sudan in which exposure to secondhand smoking outside the home was found to be associated with current cigarette smoke [9, 21]. At the same time, our findings contrast with a Madagascan study in which secondhand smoke inside the home was associated with noncigarette tobacco product use [9] and a Congolese study in which having a parent or friend who smokes was associated with using a smokeless tobacco [8]. Our findings highlight the strong influence of familial and outdoor smoking on adolescents' cigarette smoking behaviour, as reported in previous studies [28, 29]. Such influence on youth tobacco use is explained by the fact that adolescents tend to indulge in high-risk behaviours practised by their parents, siblings, or community members because they consider it normal [30]. Our finding suggests that tobacco prevention and control interventions targeting adolescents in Comoros should design educational campaigns for parents and other household members. Such a campaign should inform household members of the extent of their influence on their adolescent smoking behaviour and provide ways to prevent adolescents from using tobacco products. The influence of secondhand smoke outside the home on cigarette smoking among adolescents in our study may be due to the ineffective implementation of the law prohibiting tobacco use in public places and workplaces in Comoros [31]. The weak implementation of smoke-free laws has been attributed to inconsistencies and contrasting provisions within tobacco control laws in Comoros [18, 31]. For instance, based on the current provisions, it is difficult to ascertain whether smoking is prohibited in primary/secondary schools, shops, and Casinos [31]. Moreover, there is no clear indication of what agency is responsible, and there is no clear duty to enforce the smoke-free law [31]. Going forward, smoke-free tobacco laws need to be revised to remove any ambiguity in their interpretation. In addition, in line with article 8 of the WHO-FCTC [32], the legislation should indicate the agency responsible for enforcing the law. Furthermore, the ineffective implementation of tobacco laws in Comoros may be due to the lack of follow-up in the implementation of health policies by successive governments due to political instability in the country, and sometimes variability in the implementation of health policies between the central government in Grande Comore and the local semiautonomous governments in the other islands (Anjouan and Moheli), which may be due to the differences in their political ideologies [33]. Further studies are needed to explore the impact of previous political insecurity and instability in the implementation of tobacco laws in Comoros.

Consistent with other studies and similar studies conducted in other developing countries [7–10, 21, 34], exposure to tobacco industry promotion was significantly associated with cigarette smoking and noncigarette tobacco use in our study. The increased exposure of adolescents to tobacco industry promotion and its association with tobacco use may be due to the increased penetration of tobacco companies into the Comorian market, which may be attributed to low tobacco taxes, partial ban on tobacco promotion, and weak regulation of antitobacco promotion laws. Previous research has demonstrated that countries with less comprehensive and weak tobacco promotion, advertisement, and sponsorship regulations have a higher rate of adolescent tobacco use compared to those with comprehensive and stricter laws, which are fully implemented [35, 36]. Our finding further supports the link between tobacco product promotion and adolescent tobacco use. Currently, there are ambiguities in the interpretation of Comoros's tobacco promotion and advertisement laws, and these legislations are not strictly in line with WHO-FCTC article 13 guidelines [31]. The law allows tobacco product sales via the Internet and point of sale, advertisements and promotions, and tobacco product display. In addition, the law does not address vending machine sales of tobacco products, does not explicitly address cross-border advertising broadcast from outside of Comoros, and does not address retailer incentive programs [31]. Comoros' weak and ambiguous tobacco advertisement and promotion laws provide an opportunity for the tobacco industry to identify loopholes in current legislation or find innovative ways to promote their products among adolescents. A case in point is the use of the Internet to promote and sell their products. Compared with other exposure channels, the Internet has been reported to be a leading conduit used by tobacco companies to promote, advertise, and sell their products to adolescents in developed countries [37, 38] and this may explain the insignificant association observed between exposure to smoking media messages and cigarette use and noncigarette use in our study. Exposure to tobacco promotion via the Internet has been associated with traditional and alternative forms of tobacco use [38, 39]. Currently, it is unknown whether such exposure channels (e.g., Internet and point of sales) are widely used by tobacco companies in Comoros and that influence adolescent tobacco use among adolescents. Further studies need to explore this area of enquiry. Given the adverse health effects of tobacco use and the increase in tobacco-related chronic conditions in Comoros, it is important that the existing tobacco industry promotion, advertisement, and sponsorship laws are revised and implemented consistent with WHO-FCTC article 13 guidelines [40]. Such interventions will help prevent adolescents from becoming adult smokers.

Consistent with previous systematic reviews [41], we observed that adolescents who were not exposed to antismoking education in their schools were less likely to be current cigarette and noncigarette tobacco users. Our finding suggests that antismoking education is either not done in schools in Comoros or if it is done has not been effective in preventing adolescents from using tobacco products. Previous systematic reviews have reported that school-based tobacco prevention interventions have no effect on adolescent tobacco use [41, 42]. The ineffectiveness of tobacco prevention interventions may be due to the strong peer and family influence as it has been previously reported in other studies [43, 44]. In order to archive an impact, school-based tobacco prevention interventions such as antitobacco educational programs should be vigorous and integrated within the country school curriculum, and such an approach should be linked with family and community tobacco use prevention programs for it to have a long-term impact to prevent adolescent tobacco use [44, 45].

It is important for readers to bear in mind the following limitations when interpreting our findings. Given the cross-sectional design employed in this study, no causal relationship can be made between the outcome and independent variables. Also, the study is prone to recall bias, given that responses were based on participant self-reports. In addition, our findings are not representative of all adolescents in Comoros as only school-going adolescents were included in the study. Only individual-level measures were used based on the GYTS questionnaire. Other variables relating to national tobacco control programs or policies in Comoros that could have influenced tobacco use among adolescents were not included. Furthermore, we could not determine whether adolescent tobacco use behaviour varied in the three islands that constitute Comoros and the association between tobacco use and peer smoking as data on peer smoking was not provided in the dataset. Nonetheless, this study is the first study that examines the prevalence and correlates of tobacco use among school-going adolescents in Comoros using the most recent Comoros GYTS dataset and provides the baseline insight for future studies into tobacco use behaviour in Comoros.

## 5. Conclusion

Our study finds that one in seven school-going adolescents in Comoros smokes cigarettes, with a significant gap between males and females. Also, in our study, approximately one in 20 school-going adolescents uses noncigarette tobacco ?A3B2 show $132#?>products, but no significant gender gap exists. Exposure to secondhand smoke in and outside the house was significantly associated with the current use of cigarettes. Irrespective of gender, exposure to tobacco industry promotion was significantly associated with the current use of noncigarette tobacco products among school-going adolescents in Comoros. In addition, adolescents who received antismoking education in schools in our study were less likely to be current noncigarette tobacco users.

Our findings suggest the need to implement the following recommendations to help to prevent and reduce tobacco use among school-going adolescents in Comoros.

- In addition to cigarettes, the adverse effects of noncigarette tobacco products should be part of tobacco control interventions such as public education and advocacy strategies targeting adolescents in Comoros
- Tobacco prevention and control interventions targeting adolescents in Comoros should design educational campaigns for parents and other household members. Such a campaign should inform household members of the extent of their influence on their adolescent smoking behaviour and provide ways how they can prevent adolescents from smoking tobacco
- Tobacco smoke-free laws need to be revised to remove any ambiguity in their interpretation and implemented in accordance with the WHO-FCTC article 8 guidelines
- Existing tobacco industry promotion advertisement and sponsorship laws need to be revised and implemented consistent with WHO-FCTC article 13 guidelines. Such interventions will help prevent adolescents from becoming adult smokers
- School-based tobacco prevention interventions integrated within the country school curriculum, and such an approach should be linked with family and community tobacco use prevention programs

## Figures and Tables

**Table 1:** Study measures, survey items with responses, 2015 Comoros Global Youth Tobacco Survey (GYTS).

Study measure	GYTS survey items	GYTS item responses	Dichotomized measure	
Dependent variable: current cigarette use	During the past 30 days, on how many days did you smoke cigarettes?	0 days 1 or 2 days 3 to 5 days 6 to 9 days 10 to 19 days 20 to 29 days All 30 days	No-0 days Yes-1 or more days	
Current use of noncigarette tobacco products	During the past 30 days, did you use any form of smoked tobacco products other than cigarettes (such as cigar, pipe, water pipe, and shisha)?	Yes No	Yes No	
	During the past 30 days, did you use any form of smokeless tobacco products (e.g., chewing tobacco, snuff, and dip)?	Yes No	Yes No	
Independent variables				
Age	How old are you?	11 years old or younger 12 years old 13 years old 14 years old 15 years old 16 years old 17 years old or older	≤12years 13-15 years ≥16 years	
Sex	What is your sex?	Male Female	Male Female	
Grade/form	In what grade/form are you?	6 eme 5 eme 4 eme 3 eme	6 eme 5 eme 4 eme 3 eme	
Money to be spent on average week	During an average week, how much money do you have that you can spend on yourself, however you want?	I usually do not have any spending money Less than 500 Fr [500-1000] [1001-2500] [2501-5000] [5001-7500] [7501 and +	No money- I usually do not have any spending money Have money- any other responses for any of the remaining seven items	
Parental smoking	How often do you see your father (stepfather or mother's partner) smoking in your home?	Do not have/do not see this person About every day Sometimes Never	No- do not have/do not see this person and never Yes- about every day and sometimes	No- no for both dichotomized items
	How often do you see your mother (stepmother or father's partner) smoking in your home	Do not have/do not see this person About every day Sometimes Never	No- do not have/do not see this person and never Yes- about every day and sometimes	Yes - for both dichotomized items
SHSb exposure in your home	During the past 7 days, on how many days has anyone smoked inside your home, in your presence?	0 days 1 to 2 days 3 to 4 days 5 to 6 days 7 days	No-0 days Yes-1 to 2 days 3 to 4 days 5 to 6 days 7 days	
SHSb exposure outside home	During the past 7 days, on how many days has anyone smoked in your presence, inside any enclosed public place, other than your home (such as office, school, shops, restaurants, cinemas, and night club)?	0 days 1 to 2 days 3 to 4 days 5 to 6 days 7 days	No-0 days Yes-1 to 2 days 3 to 4 days 5 to 6 days 7 days	No- no for all three dichotomized items Yes - for all three dichotomized items
	During the past 7 days, on how many days has anyone smoked in your presence, inside any public transportation vehicles, such as trains, buses, or taxicabs?	I did not use public transportation during the past 7 days I used public transportation, but no one smoked in my presence 1 to 2 days 3 to 4 days 5 to 6 days 7 days	No- “I did not use public transportation during the past 7 days” and “I used public transportation, but no one smoked in my presence” Yes-1 to 2 days 3 to 4 days 5 to 6 days 7 days	
	During the past 7 days, on how many days has anyone smoked in your presence, at any outdoor public place (such as playgrounds, sidewalks, entrances to buildings, parks, beaches, and vehicle)?	0 days 1 to 2 days 3 to 4 days 5 to 6 days 7 days	No-0 days Yes-1 to 2 days 3 to 4 days 5 to 6 days 7 days	
Exposure to antismoking media messages	During the past 30 days, how many antismoking media messages (e.g., television, radio, billboards, posters, newspapers, magazines, and movies) have you seen?	Yes No	Yes No	No- no for both dichotomized items Yes - for both dichotomized items
Exposure to smoking media messages in the media	During the past 30 days, did you see any people using tobacco on TV, in videos, or in movies?	I did not watch TV, videos, or movies Yes No	Yes = “yes” No = “I did not watch TV, videos, or movies” and “no”	
Favour toward smoking ban	Are you in favour of banning smoking inside enclosed public places (such as schools, shops, restaurants, supermarket, shopping centers, movie theaters, and youth homes ...)?	No Yes	No Yes	
	Are you in favour of banning smoking at outdoor public places (such as stadium, public place, recreation areas, sidewalks, entrances to buildings, and beaches)?	No Yes	No Yes	
Knowledge about harmful effects of SHS	Do you think the smoke from other people's cigarettes is harmful to you?	Definitely not Probably not Probably yes Definitely yes	No “definitely not” for both items Yes - any other responses For either item	
Tobacco industry promotion	Do you have something (t-shirt, pen, backpack, etc.) with a cigarette brand logo on it?	No Yes	No Yes	No- no for both dichotomized items
	Has a cigarette representative ever offered you a free cigarette?	No Yes	No Yes	Yes - for both dichotomized items
Antismoking school education	During the past 12 months, were you taught in any of your classes about the dangers of tobacco use?	No I do not know Yes	No- no I do not know Yes- yes	

**Table 2:** Characteristics of the study population, Comoros Global Youth Tobacco Survey, 2015 (*N* = 2,810).

	Unweighted count	Weighted %
*Age*		
≤12 years	276	9.5
13-15 years	1551	55.3
≥16 years	938	35.2
*Sex*		
Male	1310	48.1
Female	1479	51.9
*Grade*		
6 eme	954	31.5
5 eme	665	25.1
4 eme	630	21.7
3 eme	546	21.8
*Possession of average spending money/week*		
I have	1976	70.2
I do not have	834	29.8
Parental smoke (yes)	584	21.2
SHS exposure inside home (yes)	793	28.8
SHS exposure outside home (yes)	1636	59.6
Exposure to smoking media messages (yes)	1213	44.3
Exposure to antismoking media messages (yes)	1373	50.6
Exposure to tobacco industry promotion (yes)	543	21.5
Knowledge about harmful effects of SHS(yes)	1918	69.7
School antismoking education (yes)	669	25.3
Favour toward smoking ban indoor and outdoor (yes)	2041	74.6

**Table 3:** Prevalence of current cigarette smoking and noncigarette tobacco product use among male and female adolescents in Comoros, Global Youth Tobacco Survey, 2015 (*N* = 2,810).

Characteristic *n* (%^a)^	Current cigarette smoker			Current user of noncigarette tobacco products		
	Total *n* (%^a^) 400(14.3)	Male *n* (%^a^) 246(18.5)	Female *n* (%^a^) 143(9.9)	Total *n* (%^a^) 145(5.8)	Male *n* (%^a^) 81(6.7)	Female *n* (%^a^) 64 (4.9)
*Age*						
≤12years	17(4.2)	9(3.5)	7(4.9)	7(5.0)	4(5.0)	3(5.0)
13-15 years	209 (53.4)	121(48.9)	85(61.9)	66(45.4)	35(43.4)	31(48.6)
≥16 years	161(42.5)	113(47.6)	45(33.2)	65(49.6)	37(51.7)	27(46.3)
*Grade*						
6 eme	142 (33.3)	97 (35.8)	41 (28.5)	42 (25.7)	29 (31.6)	12 (17.3)
5 eme	87 (23.9)	45 (21.6)	38 (26.4)	30 (21.5)	15 (21.0)	15 (22.3)
4 eme	93 (23.1)	63 (25.2)	29 (20.1)	29 (19.2)	17 (20.5)	12 (17.7)
3 eme	69 (19.7)	37 (17.4)	32 (25.0)	44 (33.6)	19(26.9)	25 (42.7)
*Possession of average spending money/week*						
I have	294 (73.6)	178(72.5)	106 (74.1)	99 (67.8)	56 (68.9)	43 (67.2)
I do not have	106 (26.4)	68 (27.5)	37 (25.9)	47 (32.2)	25 (31.1)	21 (32.8)
*Parental smoke (yes)*	145 (38.1)	85 (36.1)	53 (39.1)	38 (26.9)	18 (22.8)	20 (32.2)
SHS exposure inside home (yes)	238(60.1)	153(63.3)	82(57.1)	57(39.4)	36(45.1)	21(32.4)
SHS exposure outside home (yes)	305(78.2)	196(81.0)	101(72.7)	102(70.3)	59(73.0)	42(66.3)
Exposure to smoking media messages (yes)	203(52.9)	131(55.9)	68(48.9)	65(47.2)	37(48.9)	27(44.5)
Exposure to antismoking media messages (yes)	240(64.9)	154(67.4)	78(59.2)	75(55.5)	44(58.9)	30(50.6)
Exposure to tobacco industry promotion (yes)	142(43.4)	93(44.4)	45(40.3)	49(40.5)	30(43.8)	19(36.2)
Knowledge about harmful effects of SHS(yes)	260(68.0)	166(70.1)	86(63.7)	90(62.4)	47(59.5)	42(65.7)
School antismoking education (yes)	119(31.8)	76(32.3)	40(30.3)	51(37.4)	29(38.4)	22(36.2)
Favour toward smoking ban (yes)	284(76.6)	185(79.4)	92(72.0)	111(78.6)	64(81.8)	46(74.1)

^a^Weighted percentage. SHS: secondhand smoke. *n* = Unweighted count.

**Table 4:** Determinants of current cigarette smoking and noncigarette tobacco product use among male and female school-going adolescents. Global Youth Tobacco Survey, 2015 (*N* = 2,810).

Characteristics %^a^	Variables	Current cigarette smoker			Current user of noncigarette tobacco products		
		Total AOR (95% CI)	Male AOR (95% CI)	Female AOR (95% CI)	Total AOR (95% CI)	Male AOR (95% CI)	Female AOR (95% CI)
Sex	Male	2.24(1.39-3.63)			1.24(0.82-1.86)		
	Female	1			1		

Age	≤12years	1	1	1	1	1	1
	13-15 years	3.11 (1.53-6.35)	4.44 (1.69-11.65)	1.92 (0.85-4.34)	1.04 (0.34-3.16)	0.79 (0.14-4.35)	1.28 (0.41-3.99)
	≥16 years	4.89 (2.56-9.37)	8.83 (3.36-23.21)	2.34 (0.91-6.03)	1.84 (0.64-5.32)	1.64 (0.28-9.45)	2.13 (0.87-5.26)

Grade	6 eme	2.41 (1.36-4.27)	3.56 (1.26-10.06)	1.47 (0.59-3.63)	0.85 (0.49-1.48)	1.32 (0.71-2.43)	0.57 (0.22-1.48)
	5 eme	1.22 (0.78-1.90)	1.01 (0.50-2.05)	1.60 (0.61-4.20)	0.72 (0.43-1.22)	0.68 (0.25-1.82)	0.80 (0.38-1.70)
	4 eme	1.46 (0.81-2.62)	1.70 (0.84-3.45)	1.16 (0.40-3.31)	0.62 (0.38-1.00)	0.72 (0.32-1.65)	0.52 (0.25-1.09)
	3 eme	1	1	1	1		1

Possession of average spending money/week	I have	0.89 (0.62-1.26)	0.85 (0.57-1.27	0.96 (0.61-1.49)	0.83 (0.62-1.12)	0.66 (0.37-1.17)	1.14 (0.67-1.95)
	I do not have	1	1	1	1	1	1

Parental smoke	Yes	1.20 (0.78-1.87)	0.95 (0.58-1.54)	1.70 (0.77-3.76)	0.92 (0.40-2.11)	0.69 (0.23-2.06)	1.22 (0.52-2.86)
	No	1	1	1	1	1	1

SHS exposure inside home	Yes	3.88 (2.84-5.31)	4.90 (3.95-6.08)	2.89 (1.35-6.19)	1.47 (0.87-2.48)	1.98 (0.94-4.20)	0.97 (0.47-2.02)
	No	1	1	1	1	1	1

SHS exposure outside home	Yes	1.49 (1.08-2.03)	1.72 (1.20-2.46)	1.13 (0.60-2.13)	1.45 (0.87-2.43)	1.46 (0.89-2.41)	1.42 (0.52-3.88)
	No	1	1	1	1	1	1

Exposure to smoking media messages	Yes	0.74 (0.48-1.14)	0.59 (0.24-1.48)	1.21 (0.43-3.41)	0.69 (0.37-1.27)	0.68 (0.34-1.37)	0.77 (0.14-4.09)
	No	1	1	1	1	1	1

Exposure to antismoking media messages	Yes	1	1		1	1	1
	No	0.65 (0.47-0.90)	0.53 (0.24-1.48)	0.96 (0.33-2.81)	0.80 (0.41-1.54)	0.64 (0.32-1.29)	1.09 (0.23-5.26)

Exposure to tobacco industry promotion	Yes	2.90 (2.21-3.80)	2.66 (1.86-3.82)	3.44 (1.92-6.17)	2.58 (1.54-4.32)	2.58 (1.39-4.78)^∗^	2.54 (1.24-5.22)^∗^
	No	1	1	1	1	1	1

Knowledge about harmful effects of smoking and SHS	Yes	1	1	1	1	1	1
	No	1.12 (0.83-1.52)	1.04 (0.68-1.60)	1.37 (0.80-2.36)	1.36 (0.85-2.19)	1.75 (1.03-2.99)^∗^	1.03 (0.52-2.05)

School antismoking education	Yes	1	1	1	1	1	1
	No	0.97 (0.76-1.22)	1.04 (0.68-1.59)	0.84 (0.55-1.29)	0.52 (0.32-0.85)	0.51 (0.29-0.88)	0.56 (0.23-1.44)

Favour toward smoking ban	Yes	1	1	1	1	1	1
	No	1.16 (0.80-1.67)	0.99 (0.65-1.52)	1.58 (0.81-3.08)	0.90 (0.56-1.45)	0.68 (0.26-1.78)	1.26 (0.90-1.77)

SHS: secondhand smoke. ^∗^*p* < 0.05.

## Data Availability

The dataset informing the findings of this study is freely available via the WHO NCD Microdata Repository https://extranet.who.int/ncdsmicrodata/index.php/catalog/123.

## Abbreviations

CI: Confidence interval
FCTC: Framework Convention on Tobacco Control
GYTS: Global Youth Tobacco Survey
GTS: Global Tobacco Surveillance
OR: Odds ratio
SHS: Secondhand smoke
SPSS: Statistical Package for the Social Sciences
WHO: World Health Organization.

## Supplementary Materials

Supplementary 1 Supplementary file 1: STROBE checklist for cross-sectional studies.
