# Supplementary material for: Tobacco Use among School-Going Adolescents in Comoros: A Secondary Analysis of the 2015 Comoros Global Youth Tobacco Survey
Source: Biomed Res Int. 2022 Mar 8;2022:7134340. doi: 10.1155/2022/7134340 (PMC8923772; doi:10.1155/2022/7134340)
Supplement: Supplementary 1 — Supplementary file 1: STROBE checklist for cross-sectional studies. [file 7134340.f1.pdf]

STROBE Statement—Checklist of items that should be included in reports of ***cross-sectional studies***

	Item No	Recommendation
**Title and abstract**	1	(*a*) Indicate the study's design with a commonly used term in the title or the abstract *See page 1*
		(*b*) Provide in the abstract an informative and balanced summary of what was done and what was found. *See page 2*
**Introduction**		
Background/rationale	2	Explain the scientific background and rationale for the investigation being reported. *See page 4*
Objectives	3	State specific objectives, including any prespecified hypotheses. *See page 5*
**Methods**		
Study design	4	Present key elements of study design early in the paper. *See page 5*
Setting	5	Describe the setting, locations, and relevant dates, including periods of recruitment, exposure, follow-up, and data collection. *See page 6*
Participants	6	(*a*) Give the eligibility criteria, and the sources and methods of selection of participants. *See page 6*
Variables	7	Clearly define all outcomes, exposures, predictors, potential confounders, and effect modifiers. Give diagnostic criteria, if applicable. *See page 6&7*
Data sources/ measurement	8*	For each variable of interest, give sources of data and details of methods of assessment (measurement). Describe comparability of assessment methods if there is more than one group *See page 6&7*
Bias	9	Describe any efforts to address potential sources of bias. *See page 7*
Study size	10	Explain how the study size was arrived at. *See page 6*
Quantitative variables	11	Explain how quantitative variables were handled in the analyses. If applicable, describe which groupings were chosen and why. *See page 7*
Statistical methods	12	(*a*) Describe all statistical methods, including those used to control for confounding. *See page 7*
		(*b*) Describe any methods used to examine subgroups and interactions. *See page 7*
		(*c*) Explain how missing data were addressed. *See page 7*
		(*d*) If applicable, describe analytical methods taking account of sampling strategy. *See page 7*
		(*e*) Describe any sensitivity analyses. *Not applicable*
**Results**		
Participants	13*	(a) Report numbers of individuals at each stage of study—eg numbers potentially eligible, examined for eligibility, confirmed eligible, included in the study, completing follow-up, and analysed. *See pages 7&8*
		(b) Give reasons for non-participation at each stage. *Not applicable*
		(c) Consider use of a flow diagram. *Not applicable*
Descriptive data	14*	(a) Give characteristics of study participants (eg demographic, clinical, social) and information on exposures and potential confounders. *See pages 7,8&9*
		(b) Indicate number of participants with missing data for each variable of interest. *Not applicable*
Outcome data	15*	Report numbers of outcome events or summary measures. *See pages 7,8&9*
Main results	16	(*a*) Give unadjusted estimates and, if applicable, confounder-adjusted estimates and their precision (eg, 95% confidence interval). Make clear which confounders were adjusted for and why they were included. *See pages 7,8&9*

		(*b*) Report category boundaries when continuous variables were categorized. *See pages 7,8&9*
		(*c*) If relevant, consider translating estimates of relative risk into absolute risk for a meaningful time period. *Not applicable*
Other analyses	17	Report other analyses done—eg analyses of subgroups and interactions, and sensitivity analyses. *See pages 7,8&9*
**Discussion**		
Key results	18	Summarise key results with reference to study objectives. *See pages 9,10*
Limitations	19	Discuss limitations of the study, taking into account sources of potential bias or imprecision. Discuss both direction and magnitude of any potential bias. *See page 13*
Interpretation	20	Give a cautious overall interpretation of results considering objectives, limitations, multiplicity of analyses, results from similar studies, and other relevant evidence. *See pages 9,10,11&12*
Generalisability	21	Discuss the generalisability (external validity) of the study results. *See page 14*
**Other information**		
Funding	22	Give the source of funding and the role of the funders for the present study and, if applicable, for the original study on which the present article is based. *See page 16*

*Give information separately for exposed and unexposed groups.

Note: An Explanation and Elaboration article discusses each checklist item and gives methodological background and published examples of transparent reporting. The STROBE checklist is best used in conjunction with this article (freely available on the Web sites of PLoS Medicine at http://www.plosmedicine.org/, Annals of Internal Medicine at http://www.annals.org/, and Epidemiology at http://www.epidem.com/). Information on the STROBE Initiative is available at www.strobe-statement.org.
